# Feasibility and Acceptability of a Digital Health Portal to Improve HIV Care Engagement Among Kenyan Youth: Mixed Methods Study

**DOI:** 10.2196/59661

**Published:** 2025-07-07

**Authors:** Eric Nturibi, Jared Mecha, Florence Kaara, Faith Musau, Christine Mwangi, Elizabeth Kubo, Albert Orwa

**Affiliations:** 1 Centre for Health Quality and Innovation, Department of Clinical Medicine and Therapeutics, University of Nairobi, P.O. Box 30197, Nairobi, 00100, Kenya, 254 722758767

**Keywords:** HIV, online portal, antiretroviral, STI, STD, sexual, portal, young adult, ART, preference, engagement, experience, attitude, opinion, perception, perspective, survey, questionnaire, Africa

## Abstract

**Background:**

Adolescents and young people aged 15‐24 years in Kenya bear a disproportionate burden of HIV, necessitating innovative, youth-friendly approaches to improve care engagement. Digital solutions such as patient health portals (PHPs) offer scalable ways to enhance access, understanding, and support.

**Objective:**

This study aims to evaluate the feasibility and acceptability of a customized PHP tailored for Kenyan adolescents and young people living with HIV, and to explore participant preferences for features, content delivery modes, and privacy concerns.

**Methods:**

A cross-sectional, mixed methods feasibility study was conducted among 163 adolescents and young people recruited during psychosocial support group meetings across seven high-volume clinics in Kiambu and Kirinyaga counties in August 2021. Participants interacted with a prototype PHP through guided demonstrations and completed structured questionnaires assessing health literacy, digital readiness, feature preferences, emotional needs, and data security perceptions. Quantitative data were analyzed using descriptive and bivariate statistics. While qualitative data were also collected to inform portal development, this paper focuses exclusively on quantitative findings.

**Results:**

The median age was 18 (IQR 15‐24) years, with 60% (98/163) female participants. Most participants were students (73/163, 45%) and had secondary-level education (83/163, 51%). Approximately 17% (27/163) participants reported difficulty understanding written medical information. A large majority (146/160, 91%) expressed interest in using a web-based portal, with 78% (124/159) participants rating it as easy to use. Weekly use was anticipated by 56% (90/161) of participants. Top-rated features included appointment scheduling (129/163, 79%), access to test results (129/163, 79%), and communication with the doctor (117/163, 72%). Emotional health tools like a mood tracker and PHQ-9 (Patient Health Questionnaire-9) screening were highly valued, with 59% (92/156) reporting difficulties coping emotionally with HIV. Concerns about data privacy were minimal, and 62% (99/159) were willing to share access with a trusted family member. No statistically significant associations were observed between portal preference and age, sex, education level, or employment status.

**Conclusions:**

The study demonstrates the strong feasibility and acceptability of a digital health portal among Kenyan adolescents and young people. High readiness for digital health, combined with clearly expressed content and feature preferences, underscores the potential for such tools to improve engagement in HIV care. These findings support the need for co-designed, youth-centered digital interventions that address both medical and psychosocial needs in resource-limited settings.

## Introduction

The patient health portal (PHP) study, a stepped wedge cluster randomized controlled trial conducted between May 2022 and December 2023, evaluated the effectiveness of a tailored web-based health portal in fostering patient engagement among adolescents and young people aged 15 to 24 years. Detailed in our study protocol, the study reflects an innovative approach to address the unique needs of this demographic, who often face barriers to consistent health care access and adherence [1].

Central to the design of the PHP was the Capability, Opportunity, Motivation, and Behavior model, a framework developed by Michie et al [2] to conceptualize behavior as a dynamic interplay between capability (the possession of knowledge and skills required to perform an action), opportunity (the external conditions that enable or prompt behavior), and motivation (reflexive and automatic processes that drive actions). The Capability, Opportunity, Motivation, and Behavior model’s versatility has made it a cornerstone in designing effective health interventions, particularly for identifying and addressing barriers to behavior change [2].

**Figure 1.**
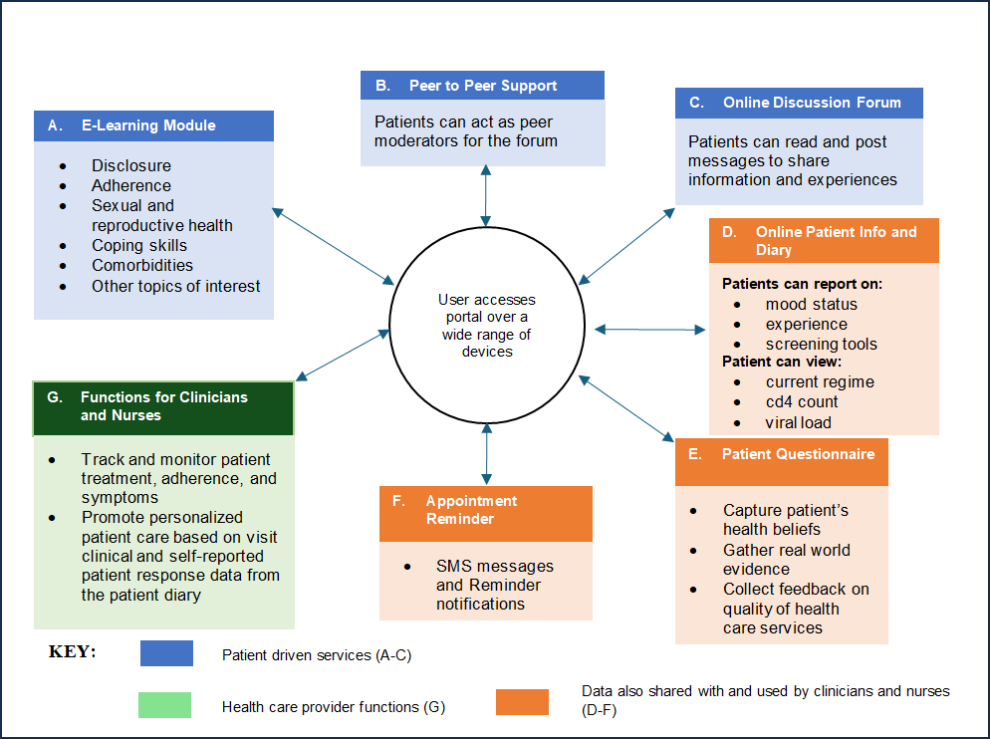
Schematic overview of the proposed digital health portal for adolescents and young people living with HIV.

The PHP portal featured an array of user-focused tools, including an e-learning module, a peer support forum, access to patient health information, a symptom diary, questionnaires, appointment reminders, and provider-specific functionalities (Figure 1).

Delivered via internet-enabled smartphones, the portal seamlessly integrates with key elements of the patient’s electronic medical record, ensuring a personalized and cohesive health care experience.

The development of the web-based health portal was motivated by the pressing need to address the disproportionate burden of HIV among young people, who account for a significant share of new infections and HIV-related deaths globally. Leveraging the readiness of adolescents and young people to adopt internet-based digital health solutions, this initiative aimed to bridge critical gaps in health care delivery [3,4]. The portal was integrated into a large-scale antiretroviral therapy program in Kenya, which, as of September 30, 2021, had provided antiretroviral therapy to 38,079 individuals living with HIV, including 3459 adolescents and young people, across 78 public health facilities (program data).

The PHP study was conducted in Kiambu and Kirinyaga counties, which represent distinct socioeconomic and epidemiological contexts for addressing HIV. Kiambu, a predominantly urban and peri-urban county, had an HIV prevalence of 4.0% among adults and 1.8% among youth aged 15‐24 years by the end of 2019 and had recorded approximately 510 new infections and 230 AIDS-related deaths. While Kiambu benefits from a thriving economy driven by agriculture, industrialization, and services, significant health care challenges persist in its informal settlements.

In contrast, Kirinyaga, a largely rural county, reported an HIV prevalence of 3.2% among adults and 1.4% among youth aged 15‐24 years, with 300 new infections and 160 AIDS-related deaths over the same period. The county’s economy relies heavily on tea, coffee, and subsistence farming, yet health care access remains constrained by economic vulnerabilities. Nationally, as of 2019, Kenya reported an HIV prevalence of 4.5% among adults, with 41,416 new infections and 21,000 AIDS-related deaths [5].

To determine the feasibility of the PHP study, we investigated smartphone ownership and internet use patterns (separate manuscript), and gathered end user feedback on the portal’s proposed features. The study objectives were designed to comprehensively assess the portal’s potential impact. Specifically, the questionnaire sought to (1) characterize the sociodemographic profiles of adolescents and young people aged 15‐24 years attending HIV care clinics; (2) evaluate readiness to adopt a web-based health portal; (3) gather and prioritize feature preferences to tailor the portal to user needs; and (4) identify specific life-stage domains critical for inclusion in the portal’s content.

## Methods

### Study Setting and Recruitment Procedures

#### Study Context

The study was implemented across seven high-volume public HIV care facilities located in Kiambu and Kirinyaga counties. These sites were intentionally selected for their large youthful populations, well-established psychosocial support programs, and participation in national adolescent-centered initiatives such as Operation Triple Zero (OTZ). The OTZ initiative targets adolescents and young people aged 10‐24 years and promotes HIV care adherence through a focus on achieving “zero missed visits, zero missed drugs, and zero viral load”.

Participant recruitment was embedded within triannual psychosocial support group (PSSG) meetings held during the August 2021 school holiday. Eligible individuals, identified through program rosters and clinic records, were aged 15‐24 years, enrolled in HIV care at one of the study clinics, and attended the PSSG session. Attendees were informed about the study through brief plenary announcements during the morning session. Interested individuals were then invited to designated study booths for further explanation.

Study staff provided oral and written information in English, Swahili, or Sheng (local slang), and obtained verbal and written consent. For minors aged 15‐17 years, assent was obtained alongside parental/guardian consent, consistent with ethical guidelines. Participation was voluntary, and no material or financial incentives were offered beyond routine PSSG refreshments and transport reimbursements. Individuals who chose not to participate in the focused group discussions (FGDs) were encouraged to engage in alternative PSSG activities.

#### Study Procedures

##### Overview

The PSSGs provided a strategic setting for introducing the proposed health portal. A designated project representative delivered step-by-step slide presentations, using screenshots to demonstrate the portal’s login process, core functionalities, navigation tools, and operational features. A total of seven FGDs were conducted—one at each participating facility—comprising 20 to 25 participants each. Groups were stratified based on key attributes such as age, viral suppression status, and treatment duration to allow targeted feedback aligned with programmatic priorities.

Verbal consent for audio recording and note-taking was obtained, and participants formalized their agreement by signing a combined “attendance and consent form”.

The FGDs followed a structured format, guided by a pretested questionnaire distributed to all participants. Facilitators introduced each question in English and, when necessary, paraphrased the content in Swahili or local slang to ensure comprehension and inclusivity. Participants were encouraged to engage in open discussions, and adequate time was allocated for them to document their responses. Secondary facilitators provided assistance to those requiring additional support, ensuring comprehensive engagement before progressing to subsequent sections. Key discussion points were recorded by a designated note-taker, and all completed questionnaires were collected at the conclusion of each session.

The questionnaire was thoughtfully developed by the project’s psychosocial support team, drawing on over a decade of experience conducting PSSG sessions with adolescents and young people living with HIV. Its design incorporated insights from Kenya’s National HIV Guidelines (2022) [6] OTZ program tools, and validated instruments including PHQ-9 (Patient Health Questionnaire-9) and CRAFFT (Car, Relax, Alone, Forget, Friends, Trouble) [7,8].

The final version of the questionnaire was organized into four distinct sections to ensure clarity and comprehensiveness.

##### Demographics

This section captured detailed sociodemographic characteristics of participants to understand their backgrounds and how these might influence engagement with the proposed digital health portal. Data collected included age, recorded in years and calculated from date of birth, to represent younger adolescents aged 15 to 19 years and older youth aged 20 to 24 years; sex, documented to analyze gender distribution and differences in preferences and readiness for digital solutions; educational status, categorized as primary, secondary, postsecondary such as diploma or undergraduate, or no formal education to assess the impact of literacy on portal use; and employment status, classified as student, unemployed, casual worker, self-employed, or formally employed to examine socioeconomic factors affecting access to digital tools.

##### Health Literacy and Portal Readiness

This part of the questionnaire evaluated the participants’ ability to comprehend and use medical information, as well as their willingness to adopt the proposed digital portal. Key aspects included health literacy, where participants rated their ability to understand written medical information and instructions, highlighting gaps in traditional approaches; satisfaction with existing methods, measuring preferences and perceived limitations in current health education methods such as face-to-face sessions and printed materials; and portal readiness, assessing familiarity with smartphones and internet use, impressions of the portal prototype presented during sessions, and confidence in navigating its features independently. The findings highlighted the participants’ current health literacy levels and openness to adopting innovative digital health solutions, forming a baseline for designing user-centered features.

##### Portal Features and Privacy Concerns

This section of the questionnaire examined participants’ preferences for portal functionalities, concerns about data privacy and security, and anticipated use patterns. Participants ranked features such as appointment management, access to medical records, test results, symptom diaries, educational content, and communication tools with health care providers. They also shared views on data security, emphasizing confidentiality and their willingness to share portal access with family members or caregivers. Additionally, expectations regarding the frequency of portal use, whether daily, weekly, or as needed, and the specific circumstances under which the portal would be used were recorded. These insights informed the prioritization of features that balance functional needs with the sensitive nature of managing personal health information.

##### Content Relevance

This section identified the life-stage domains most critical to participants and assessed their preferred methods for receiving health-related information. Participants prioritized topics such as disease progression, symptom management, psychosocial challenges, nutrition, physical activity, financial literacy, and social support resources. Preferred delivery methods included videos, SMS updates, pictorials, question-and-answer forums, storytelling, and animations, evaluated for their effectiveness in presenting information in an engaging and accessible manner.

The questionnaire was pretested at nonstudy sites over 2 months, incorporating feedback from peer mentors and small youth groups during OTZ clinic days to refine clarity, validity, and reliability. Completion time ranged from 30 to 45 minutes.

This study used a mixed methods design, integrating quantitative survey data with qualitative insights from FGDs to enhance understanding of participant preferences. Quantitative reporting in this manuscript follows the STROBE (Strengthening the Reporting of Observational Studies in Epidemiology) guidelines. Qualitative procedures were guided by the SRQR (Standards for Reporting Qualitative Research) framework. This paper primarily reports the quantitative findings.

### Ethical Considerations

This study was conducted as part of a quality improvement initiative under the University of Nairobi CRISSP+ (Central Kenya Response: Integration, Strengthening, and Sustainability - Plus) Project. The project received ethics approval from the AMREF Health Africa Ethics and Scientific Review Committee (ESRC), with final approval granted in April 2022 (Ref: ESRC P1164/2022). All procedures were carried out in accordance with institutional and national ethical standards, and conformed to the principles outlined in the Declaration of Helsinki. As this was a quality improvement initiative involving minimal risk, and consistent with ESRC guidance, a waiver of written informed consent was granted. Nonetheless, participants were informed about the purpose of the initiative, assured of confidentiality, and given the option to decline participation without penalty.

### Sample Size Estimation

This feasibility study uses a mixed methods approach, incorporating both quantitative and qualitative components. The larger trial for which this study provides preliminary data has a computed sample size of 480 participants, with follow-up planned for 12 months [1].

For the quantitative component of this study, Cochran formula was used to determine the appropriate sample size [9]:


$$n=\frac{Z^{2}.p.\left(1-p\right)}{e^{2}}$$

where:

- Z=1.96 (z score for 95% confidence)
- p=0.5 (estimated proportion for maximum variability)
- e=0.1 (margin of error).

Given the finite population of 2647 young people in the study sites, the sample size was adjusted using the finite population correction:


$$n_{adjusted=\frac{n}{1+\frac{n-1}{N}}}$$

This yielded a minimum sample size of approximately 93 participants. Our survey included 163 participants.

### Analysis

The dataset underwent rigorous cleaning and coding processes prior to analysis to ensure accuracy and integrity. Univariate analyses were conducted to summarize participant characteristics and key variables of interest. Continuous variables, such as age, were described using medians and ranges, while categorical variables, such as sex, education level, and clinic type, were summarized using frequencies and percentages. The results of these univariate analyses were presented in tables and figures for clarity and ease of interpretation.

Bivariate analyses were then conducted to explore associations between participant demographic characteristics and their preferences or readiness to use the web-based health portal. The chi-square test was used to examine relationships between categorical variables, such as sex, education level, and employment status, with outcomes such as portal preference and perceived ease of use. For continuous variables like age, the mean and variance were calculated and compared across subgroups where relevant.

All statistical analyses were performed using Stata (version 13; StataCorp LP) [10].

## Results

### Background Characteristics

The study included 163 participants aged 15‐24 years from diverse settings within Kiambu and Kirinyaga counties, including urban, peri-urban, rural, and informal settlements (Table 1).

**Table 1.** Geographic and settlement profile of study participants aged 15‐24 years in Kiambu and Kirinyaga counties, Kenya (August 2021).

County and population		Participants (n=163), n (%)
Kiambu		
Urban		47 (29)
Informal settlement		16 (10)
Peri-urban		47 (29)
Kirinyaga		
Rural		53 (32)

A total of 60% (98/163) of the participants were female, 15% (24/163) of the participants attended prevention of mother to child transmission clinics, while 3% (5/163) of the participants had high viral loads. The median age was 18 years (Table 2). Educational backgrounds varied, with most participants (81/163, 51%) having completed secondary school. Most participants were students (72/163, 45%).

**Table 2.** Demographic summary of adolescents and young people living with HIV enrolled in a feasibility assessment of a proposed health portal in Kenya (August 2021).

Characteristic	Participants (n=163)
Sex (female), n (%)	98 (60)
Age (years), median (range)	18 (15-24)
Clinic type, n (%)	
Comprehensive Care Center	139 (85)
Prevention of mother to child transmission (PMTCT)	24 (15)
Education type, n (%)	
Primary	58 (36)
Secondary	83 (51)
College diploma	7 (4)
Undergraduate	15 (9)
Occupation, n (%)	
Student	73 (45)
Jobless	38 (23)
Casual	15 (9)
Self employed	29 (18)
Employed (formal)	8 (5)

### Health Literacy and Readiness to Use a Web-Based Portal

In this second section, participants were asked to assess their ability to understand medical information, readiness to adopt a web-based health portal, and the ease of use of the portal prototype. Approximately 17% (27 of 163) of respondents reported frequent difficulties in understanding written medical information (Table 3).

**Table 3.** Proportion of participants reporting challenges in understanding medical information during a portal usability study in Kenya (August 2021).

Rating	Values (n=163), n (%)
Always	18 (11)
Often	10 (6)
Sometimes	72 (44)
Occasionally	18 (11)
Never	45 (28)

A majority (146/160, 91%) expressed a preference for using a web-based health portal to manage their health (Table 4), and 78% (124 of 159) found the prototype demonstrated during the PSSG meetings to be user-friendly (Table 5).

**Table 4.** Agreement with the statement: “I would use a portal to manage my health” among young people living with HIV in Kenya (August 2021).

Rating	Values (n=160), n (%)
Strongly agree	93 (58)
Agree	53 (33)
Neutral	6 (4)
Disagree	6 (4)
Strongly disagree	2 (1)

**Table 5.** Ease of use rating for the portal prototype demonstrated during support group meetings with young people living with HIV in Kenya (August 2021).

Rating	Values (n=159), n (%)
Very easy	41 (26)
Easy	83 (52)
Somewhat difficult	13 (8)
Difficult	13 (8)
Very difficult	9 (6)

More than half of the participants (90/161, 56%) anticipated weekly use of the portal once implemented (Table 6).

Bivariate analysis using chi-square tests revealed no statistically significant associations between portal preference and demographic factors such as age (*P*=.82), sex (*P*=.62), education level (*P*=.58), or employment status (*P*=.25).

**Table 6.** Participant responses on how frequently they anticipate using the digital health portal if implemented (Kenya, August 2021).

Frequency	Values (n=161), n (%)
Daily	43 (27)
Weekly	47 (29)
Monthly	37 (23)
3 to 6 monthly	26 (16)
Rarely or not at all	8 (5)

### Portal Features and Privacy Concerns

Section 3 explored the specific features respondents desired in the portal, their concerns about privacy and security, and how they envisioned using the platform, including use frequency and access rights. Features such as appointment management, access to medical records and test results, and communication with health care providers were rated the highest (Table 7).

Additionally, the majority of respondents (105/159, 66%) indicated a willingness to grant access rights to a family member (Table 8).

**Table 7.** Feature preferences rated by participants for inclusion in a digital health portal tailored to young people living with HIV (Kenya, August 2021).

Feature	Values (n=163), n (%)
Allergy information	64 (39)
Caregiver access	64 (39)
Record treatment supporter	91 (56)
Lifestyle choices	98 (60)
Service requests	103 (63)
Sign up for reminders & updates	106 (65)
List of providers	109 (67)
Receive health information	109 (67)
Medication information	109 (67)
Communicate with peers	111 (68)
View medical records	116 (71)
Communicate with doctor	117 (72)
Test results	129 (79)
Appointments	129 (79)

**Table 8.** Participant views on sharing portal access with a trusted family member in the context of HIV care (Kenya, August 2021).

Access rights	Values (n=159), n (%)
Family member	99 (62)
No one	51 (32)
Other	9 (6)

### Content Relevance

Section 4 aimed to identify key life-stage concerns of young individuals to determine the most valuable content areas for inclusion as “health tips” and to assess preferred methods for content delivery. Participants evaluated the extent to which the portal prototype might enhance various aspects of their health care experience, including the quality of patient-provider communication, their understanding of health, sense of control, reduction of worries, satisfaction with care, and the safeguarding of data privacy and confidentiality (Table 9).

Participants then rated the importance of content related to their disease, overall health, symptomatology, progression of their condition over time, and aspects of psychosocial, physical, and financial well-being (Table 10).

**Table 9.** Perceived influence of portal use on different aspects of health care experience among Kenyan adolescents and young people (August 2021).

Domain	Rating by participants, n (%)				
Greatly improved	Slightly improved	No effect	Worsen slightly	Worsen greatly
Communication with my doctor (n=153)	113 (74)	26 (16)	12 (8)	0 (0)	2 (1)
Understanding of my health (n=152)	119 (78)	18 (12)	14 (9)	0 (0)	2 (2)
My sense of control (n=148)	104 (70)	24 (16)	16 (11)	0 (0)	4 (3)
My worries (n=149)	98 (66)	18 (12)	22 (15)	7 (5)	3 (3)
Safety of my care (n=146)	101 (69)	20 (14)	19 (13)	4 (3)	1 (1)
Satisfaction with care (n=148)	108 (73)	24 (16)	12 (8)	4 (3)	0 (0)
Overall quality of my health care (n=147)	107 (73)	28 (19)	12 (7)	0 (0)	0 (0)
Data privacy and security (n=154)	108 (70)	18 (12)	18 (12)	6 (4)	3 (2)

**Table 10.** Perceived influence of portal use on different aspects of health care experience among Kenyan adolescents and young people (August 2021).

Domain	Rating by participants, n (%)				
Very important	Important	Not really important	Unimportant	Very unimportant
Being aware of all details about my health (n=163)	130 (80)	29 (18)	2 (1)	2 (1)	0 (0)
Understand the cause of my symptoms (n=162)	100 (62)	55 (33)	6 (4)	0 (0)	0 (0)
Knowing whether I am healthy (n=161)	126 (78)	32 (20)	0 (0)	0 (0)	3 (2)
Knowing how my condition will evolve (n=161)	118 (73)	39 (24)	2 (1)	3 (2)	0 (0)
Knowing that people in my community do not discriminate me (n=161)	76 (47)	47 (29)	31 (19)	5 (3)	3 (2)
Find information that is relevant to my personal health situation (n=160)	109 (68)	42 (26)	5 (4)	3 (2)	2 (1)
Trust the reliability of the health information I find myself (n=160)	106 (66)	50 (31)	3 (3)	0 (0)	2 (1)
To be informed of factors that influence your health without realizing them (n=157)	108 (69)	36 (22)	8 (5)	5 (3)	0 (0)
To be educated about the future of my social and financial security (n=161)	106 (66)	45 (28)	5 (3)	2 (1)	3 (2)
How important is it for you to understand exactly what impact your diet has on your health? (n=161)	121 (75)	34 (21)	3 (2)	2 (1)	2 (1)
How important is it for you to understand exactly what impact your physical activity has on your health? (n=161)	98 (61)	58 (36)	2 (1)	0 (0)	3 (2)

To evaluate the utility of proposed digital tools—such as the PHQ-9 for depression screening and the CRAFFT for identifying alcohol use disorders—participants were asked to rate their emotional well-being. Of the 156 adolescents and young people living with HIV who participated, 59% reported some level of difficulty coping emotionally with their condition. Specifically, 23% found it somewhat difficult, 21% found it difficult, and 15% found it very difficult. Conversely, 30% found it easy, and 11% very easy to manage the emotional impact of their diagnosis.

Since the portal was designed to include a symptom diary and a Tuberculosis screening tool, participants were asked to rate the importance of disease symptom awareness (Table 11).

To determine the most effective format for delivering content through the portal, participants were asked to rate common delivery methods. Video, SMS, and question-and-answer formats emerged as the most preferred options, while role-playing, animations, and storytelling were the least favored (Table 12).

**Table 11.** Importance attributed to symptom awareness and monitoring in the context of HIV care among Kenyan adolescents and young people (August 2021).

Domain	Rating by participants, n (%)				
	Strongly agree	Agree	Neutral	Disagree	Strongly disagree
Sometimes I wonder how common the symptoms I have are (n=161)	56 (35)	82 (51)	16 (10)	5 (3)	2 (1)
Sometimes, I wonder whether my symptoms are severe enough to take action (n=161)	82 (51)	60 (37)	13 (8)	5 (3)	2 (1)
Sometimes, I wonder whether there are symptoms I should watch out for, so I can take actions in time (n=161)	90 (56)	61 (38)	3 (2)	5 (3)	2 (1)

**Table 12.** Preferred digital formats for health content delivery as selected by adolescents and young people participants during the feasibility study (Kenya, August 2021).

Mode	Rating by participants, n (%)				
Strongly preferred	Preferred	Neutral	Disliked	Strongly disliked
SMS (n=162)	86 (53)	52 (32)	10 (6)	10 (6)	5 (3)
Pictorials (n=155)	54 (35)	51 (33)	22 (14)	11 (7)	17 (11)
Animations (n=157)	50 (32)	44 (28)	25 (16)	19 (12)	19 (12)
Role plays (n=154)	57 (37)	32 (21)	25 (16)	22 (14)	18 (12)
Storytelling (n=155)	48 (31)	48 (31)	23 (15)	12 (8)	23 (15)
Question and answer (n=156)	84 (54)	47 (30)	11 (7)	12 (8)	2 (1)
Audio (n=157)	66 (42)	55 (35)	19 (11)	9 (6)	8 (5)
Video (n=155)	98 (63)	40 (26)	8 (5)	5 (3)	5 (3)

## Discussion

### Principal Findings

This preliminary investigation explores the perspectives of young people on a digital health portal designed to enhance sustained engagement in HIV care and improve treatment outcomes. Our findings suggest that the portal is both timely and feasible, addressing a critical gap identified in a systematic review, which noted that many mobile health interventions fall short of aligning with user preferences [11].

Participants were recruited from a range of settings—urban, peri-urban, rural, and informal settlements—capturing the diversity of clinic populations typical of sub-Saharan Africa. The gender distribution reflects the elevated HIV burden among adolescent girls and young women aged 15‐24, who remain disproportionately affected relative to their male peers [12]. Perspectives from subgroups such as antenatal clinic attendees, individuals with high viral loads, and those from varied occupational backgrounds further underscore the portal’s broad relevance and adaptability.

Approximately 60% of participants reported difficulty navigating traditional medical information formats. This finding echoes research from a high HIV-burden county in Kenya, where low functional health literacy hindered tasks like interpreting appointment cards and understanding medical forms [13,14]. These results highlight the importance of integrating audio-visual formats tailored to young users’ learning preferences, an approach reinforced by a Nairobi-based study that advocates for participatory and engaging educational methods to enhance comprehension and user involvement [15].

Participants showed high readiness to adopt the portal, with overwhelmingly positive feedback on its usability—an encouraging sign for scalability. This reception aligns with the e-commerce acceptance model, where perceived ease of use and perceived usefulness are critical determinants of technology adoption [16]. Nevertheless, nearly 20% of respondents anticipated challenges in using the portal, emphasizing the need for ongoing user feedback and iterative refinement to uphold a human-centered design approach [17].

Encouragingly, 80% of participants indicated they would engage with the portal at least once a month, offering the content management team a basis for update scheduling. Essential features—such as appointment reminders, access to medical records, and test results—were purposefully integrated to align with existing clinic electronic medical records. Notably, 62% of participants expressed willingness to share access with family members, suggesting the need for built-in guidance on disclosure practices. In similar Kenyan settings, structured disclosure services have been associated with improved viral suppression among adolescents [18].

Emotional wellness features also resonated strongly with participants. Tools such as the mood tracker, PHQ-9 questionnaire, and coping resources were particularly well-received. Their inclusion was guided by national guidelines and reinforced by previous studies demonstrating the centrality of emotional support in adolescent HIV care [6-8,19]. Likewise, the expressed interest in a “symptom diary” underscores the value of early symptom detection—especially for comorbid conditions such as tuberculosis—where mobile applications have proven effective in enhancing screening and accelerating treatment initiation [20,21].

Beyond clinical features, participants prioritized content on nutrition and physical fitness, mirroring global trends in youth health information needs [11,22]. Moreover, strong demand for content addressing social and financial security points to broader age-specific concerns, including the economic burdens of chronic disease management. These findings are aligned with initiatives like DREAMS (determined, resilient, empowered, AIDS-free, mentored, and safe), which seek to mitigate structural drivers of HIV risk—such as poverty and gender inequality—among adolescent girls and young women [23-25]. Emerging evidence continues to support the effectiveness of this integrated, multisectoral approach [26].

### Limitations

This survey, as a preliminary step to inform a controlled trial, relied on a convenience sample of 163 participants, representing approximately 6% of the target population. The use of a convenience sampling approach may limit the generalizability of the findings. As such, the perspectives of some young individuals living with HIV in Kenya or other contexts may not have been fully captured. Additionally, potential selection bias may have influenced the results, as participants attending PSSG meetings might differ systematically from those not engaged in these support structures. Future studies should consider random sampling strategies and broader recruitment frameworks to enhance external validity.

Although the study included qualitative data from FGDs, this paper focuses solely on the quantitative results. The number of FGDs conducted and their composition were aligned with programmatic groupings, and while they provided valuable design insights, thematic saturation was not explicitly assessed.

### Conclusions

This study underscores the potential benefits of the proposed web-based portal as a tool for improving HIV care among young individuals. By leveraging user-centered design principles and integrating practical, engaging features, the portal can address critical gaps in HIV care. Future developers of digital health solutions should prioritize feasibility studies to ensure interventions align with user expectations and capabilities.

## Abbreviations

CRAFFT: Car, Relax, Alone, Forget, Friends, Trouble
DREAMS: determined, resilient, empowered, AIDS-free, mentored, and safe
FGD: focused group discussion
OTZ: Operation Triple Zero
PHP: patient health portal
PHQ-9: Patient Health Questionnaire-9
PSSG: psychosocial support group
SRQR: Standards for Reporting Qualitative Research
STROBE: Strengthening the Reporting of Observational Studies In Epidemiology
